# Muir–Torre syndrome: sebaceous carcinoma concurrent with colon cancer in a kidney transplant recipient; a case report

**DOI:** 10.1186/s12882-019-1592-7

**Published:** 2019-10-29

**Authors:** Masahiro Tomonari, Mariko Shimada, Yasuyuki Nakada, Izumi Yamamoto, Munenari Itoh, Yusuke Koike, Akimitsu Kobayashi, Jun Miki, Hiroki Yamada, Takahiro Kimura, Shinya Saito, Kokichi Sugano, Shigeki Sekine, Hiroyasu Yamamoto, Akihiko Asahina, Takashi Yokoo

**Affiliations:** 1 Division of Nephrology and Hypertension, Department of Internal Medicine, The Jikei University School of Medicine, 3-25-8, Nishi-shimbashi, Minato-ku, Tokyo, 105-8461 Japan; 2 Department of Dermatology, The Jikei University School of Medicine, Tokyo, Japan; 3 Department of Urology, The Jikei University School of Medicine, Tokyo, Japan; 4 Oncogene Research Unit/Cancer Prevention Unit, Tochigi Cancer Center Research Institute, Tochigi, Japan; 5 Division of Pathology and Clinical Laboratories, National Cancer Center Hospital, Tokyo, Japan

**Keywords:** Sebaceous carcinoma, Muir–Torre syndrome, Microsatellite instability, Mismatch repair gene, Kidney transplant recipient

## Abstract

**Background:**

Sebaceous carcinoma is a rare but progressive malignant skin cancer, and the incidence is approximately five times higher in post-transplant patients than in people who have not received kidney transplants. Sebaceous carcinoma is sometimes found concurrently with visceral cancers and a genetic abnormality, Muir–Torre syndrome. We report the case of a female kidney transplant recipient with sebaceous carcinoma concurrent with colon cancer 10 years after transplantation.

**Case presentation:**

A 43-year-old woman was admitted due to a rapidly progressive tumor on her head. Histologically, the tumor was diagnosed as sebaceous carcinoma. We diagnosed her with Muir–Torre syndrome based on the following evidence: 1) high prevalence of microsatellite instability in gene locus assay, 2) absence of mismatch repair proteins in the sebaceous carcinoma on immunohistochemical analysis, and 3) a genetic mutation of 1226_1227delAG in the MSH2 exon 7 in the lesion detected by DNA sequencing analysis. Several reports have shown an association between immunosuppressive agents and latent Muir–Torre syndrome progression. Therefore, the progression of colon cancer in this case originated from her genetic mutation for Muir–Torre syndrome and long-term use of immunosuppressive agents.

**Conclusion:**

This case report not only highlights the importance of adequate diagnosis and therapy for Muir–Torre syndrome, but also suggests the further prevention of the development of malignant tumors in kidney transplant recipients. Physicians should be mindful that sebaceous carcinoma in kidney transplant recipients is highly concurrent with Muir–Torre syndrome.

## Background

Sebaceous carcinoma (SC) is a very uncommon cutaneous appendageal tumor; it accounts for 0.2–4.6% of all malignant cutaneous neoplasms, and the estimated development rate is only one to two per one million individuals per year [1]. It can arise from any sebaceous gland in the skin in the periocular area and the head and neck region. While little is known about the etiology of SC, a history of irradiation, immunosuppression, or familial retinoblastoma seem to be risk factors for its development [2]. A biopsy is essential to establish a diagnosis and differentiate it from other skin tumors, including other sebaceous neoplasms, adnexal tumors, and basal cell carcinoma [1]. The only curative treatment for SC is surgical resection, and chemotherapy and radiotherapy are optional but compensatory for advanced cases. Unfortunately, the prognosis of advanced stage SC is poor; patient survival is 50% at 5 years and 30% at 10 years [1].

Of note, occasional SC cases may be associated with Muir–Torre syndrome (MTS), a subtype of hereditary nonpolyposis colorectal cancer (HNPCC; Lynch syndrome) characterized by the association of at least one sebaceous skin tumor and at least one internal malignancy [3]. Among sebaceous neoplasms with MTS, the prevalence of SC is higher than in the general population (approximately 30%) [3]. Somatic or germline epi/genetic alteration of DNA mismatch repair (MMR) genes leads to loss of MMR function and increases lifetime risk of developing visceral tumors in MTS [3].

Here, we report a kidney transplant recipient with MTS who developed SC and colon cancer 10 years after transplantation. This case provides further insight into the association between the development of MTS/SC and receiving a transplant.

## Case presentation

**Fig. 1:**
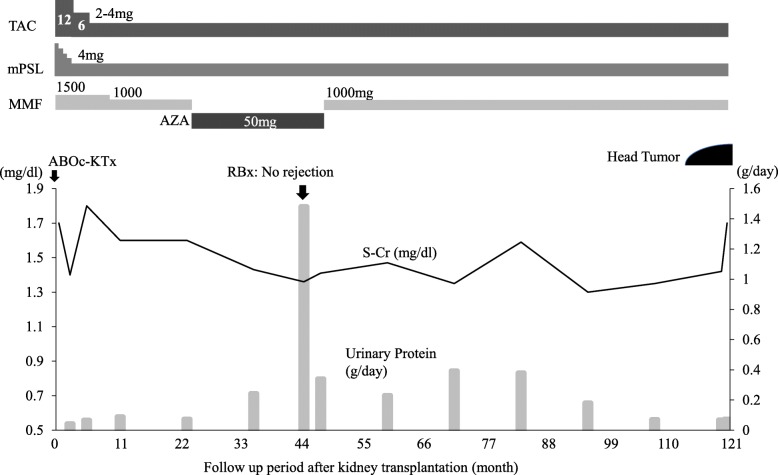
The patient’s clinical course. TAC, tacrolimus; mPSL, methylprednisolone; MMF, mycophenolate mofetil; AZA, azathioprine; ABOc-KTx, ABO-compatible kidney transplantation; RBx, renal biopsy; S-Cr, serum creatinine

**Fig. 2:**
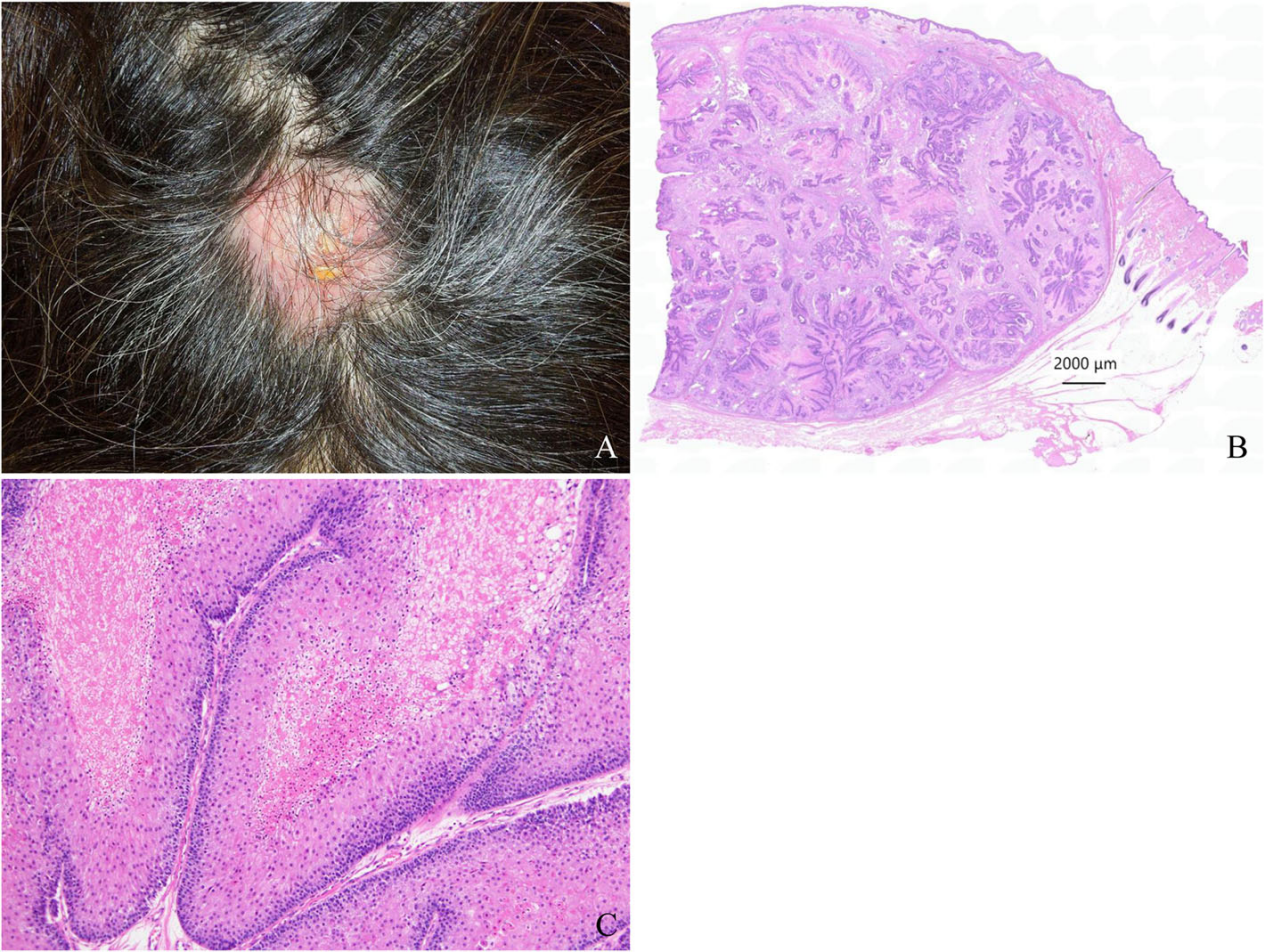
Clinical and pathological presentation of sebaceous carcinoma. **a** Sebaceous carcinoma with an axis of 4 cm on the top of the head. The tumor was soft and elastic, and the central lesion had an ulcerated surface. **b** At low magnification with hematoxylin-eosin staining, the sebaceous carcinoma was seen to be present exclusively in subcutaneous tissue and to contain scattered lobular or tubular structures within stromal tissues covered with fibrous capsules. **c** At high magnification, each lobule consisted of basaloid-like cells and sebocyte-like cells with vacuolated cytoplasm, which were partly necrotized

**Fig. 3:**
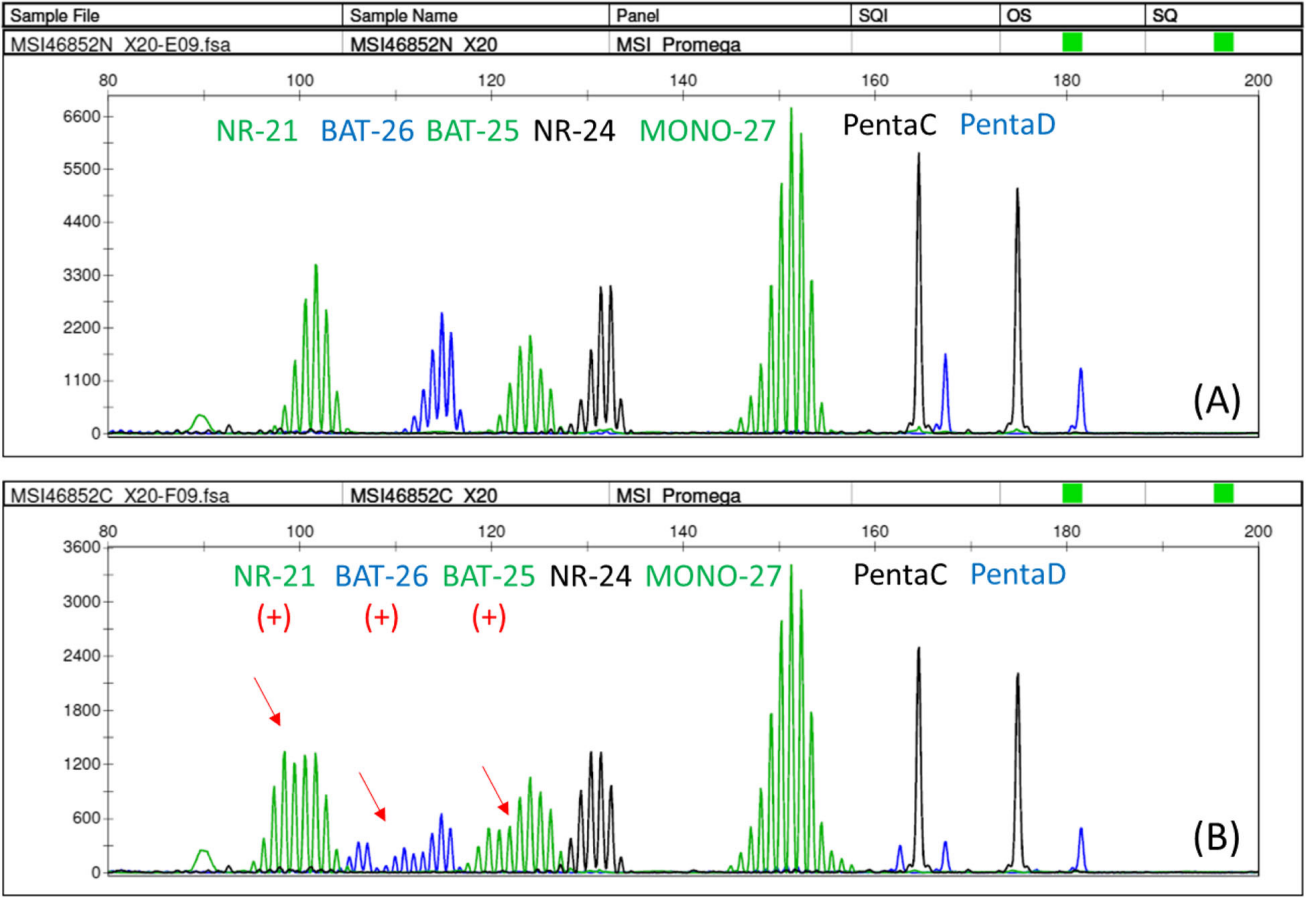
Microsatellite instability (MSI) gene locus assay using the Promega MSI Analysis System, consisting of five mononucleotide repeat markers (BAT-25, BAT-26, NR-21, NR-24, and MONO-27) and two pentanucleotide markers (Penta C and Penta D), showing MSI in three of the seven gene loci (MSI-H). **a** Control. **b** Sebaceous carcinoma in this case

**Fig. 4:**
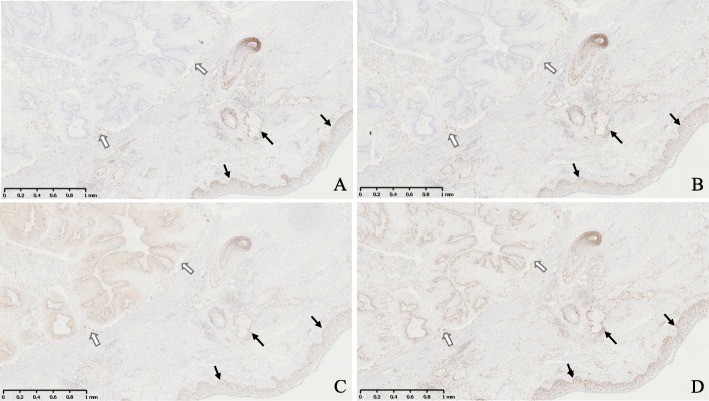
Immunohistochemical staining for DNA mismatch repair-related proteins (MMR proteins MSH2, MSH6, MLH1, and PMS2). The sebaceous carcinoma (white arrow) lacked MSH6 (**a**) and MSH2 (**b**) MMR proteins, but showed positive staining for PMS2 (**c**) and MLH1 (**d**). Normal epidermis and sebocytes (black arrow) expressed all four MMR proteins

A 43-year-old woman was admitted to our hospital because of a tumor on the back of her head. She had undergone hemodialysis due to end-stage kidney disease caused by IgA nephropathy when she was 32 years old. The next year, she received an ABO-compatible living-related kidney transplant from her father. Her allograft kidney was maintained by immunosuppressive therapy consisting of tacrolimus, mycophenolate mofetil (MMF), and methylprednisolone. After transplantation, her serum creatinine level remained stable at 1.5 mg/dL. In addition, her serum trough level of tacrolimus was mostly controlled at 3 to 5 ng/ml after transplantation. The patient’s clinical course is outlined in Fig. 1. Ten years after transplantation, she noticed a soft and compressible tumor on the left back of her head, and the major axis rapidly increased from 2 to 4 cm within 2 months (Fig. 2 A). Echography revealed the characteristics of the tumor, including border irregularity, heterogeneity, and hypervascularity, suggesting malignant properties. Surgical resection of the tumor was then performed for diagnosis and treatment. Histological findings revealed solid aggregations of neoplastic cells that varied in size in the thickness of the dermis and subcutaneous tissue exclusively (Fig. 2 B). There were many luminal structures that varied in shape inside the aggregations. Coagulative necrosis containing eosinophilic products and lipid granules in the cytoplasm of the cells was demonstrated in alveolar structures of the tumor on hematoxylin-eosin staining (Fig. 2 C). In addition, a high rate of neoplastic cells was stained by Ki-67 (MIB-1 index, 22%), diagnosing the neoplasm as SC. To diagnose the MTS, further screening of visceral tumors was performed. The examinations revealed an early-stage adenocarcinoma in the transverse colon, which was subsequently treated with endoscopic mucosal resection. To make a definite diagnosis of MTS, we performed the following examinations of the tumors: 1) MSI gene locus assay, 2) immunohistochemical staining with MMR gene proteins, and 3) DNA direct sequencing of MMR genes. We performed MSI gene locus assays with the Promega MSI Analysis System (Fig. 3), which consists of five mononucleotide repeat markers (BAT-25, BAT-26, NR-21, NR-24, and MONO-27) and two pentanucleotide markers (Penta C and Penta D). MSI in three of the seven markers was positive (MSI-H), indicating a high probability of MTS. HNPCC/MTS is induced by defective MMR due to germline mutations in any one of the DNA MMR genes. The deficiency of MMR is commonly demonstrated by the lack of any MMR proteins—MSH2, MSH6, MLH1, or PMS2—in neoplasms. The DNA MMR system mainly consists of two different heterodimers: MutSα (ex. MSH2-MSH6) and MutSβ (ex. MLH1-PMS2), and its defect, cause a dysfunction in the repair of base-base mismatches and small insertions and deletions, leading to the development of neoplasms [4]. Immunohistochemical staining demonstrated the disappearance of MMR gene proteins in neoplastic cells of both the SC and colon cancer. All four MMR gene proteins—MSH2, MSH6, MLH1, and PMS2—were positive in normal epidermis and sebocytes, while the neoplastic cells of the SC were negative for MSH2 and MSH6 (Fig. 4). The colon cancer also exhibited the significant disappearance of two MMR gene proteins (MSH2, MSH6), but normal development of MLH1 and PMS2. Furthermore, the patient’s familial history included only one brother with colon cancer, at 48 years of age. The patient’s genetic assessment using real-time PCR/direct sequencing detected a germline mutation of c.1226_1227delAG, p.Gln409ArgfsX7 in MSH2 exon 7, which was previously reported as the cause of HNPCC [5], and finally we diagnosed her with MTS. Thus, a low dose of everolimus, a mammalian target of rapamycin (mTOR) inhibitor, was administered and the serum trough level of tacrolimus dropped to 2 ng/ml at a dose of 1 mg/day to prevent further development of neoplasms due to MTS.

## Discussion and conclusions

We report an unusual case of SC complicated with colon cancer 10 years after transplantation in a female kidney recipient. Immunohistochemical and genetic findings for these malignant tumors revealed a defect of DNA MMR gene proteins, the development of MSI, and the mutation of DNA MMR genes located in MSH2, which led to a diagnosis of MTS. This case should help address the possible role of immunosuppressive agents in the development of neoplasms, and potential cancer prevention strategies in kidney transplant recipients with MTS.

Immunosuppressive agents seem to be associated with an exceptionally high rate of sebaceous neoplasms compared with the rate in the normal population. Harwood et al. reported a systemic examination of cutaneous appendageal tumors and described the high frequency of sebaceous origin and malignancy in transplant recipients compared with the general population (30% vs. 6%; *P* < 0.0001 for sebaceous origin, 43.5% vs. 4.1%; *P* < 0.0001 for malignancy) [6]. Immunosuppressive agents cause a high prevalence of MSI [7]. Generally, MSI is characterized by the accumulation of numerous mutations across the genome, mainly in repetitive sequences (microsatellites) due to impaired MMR, and is a very useful screening marker because it is found in 60% of sebaceous malignant neoplasms as compared to only 3% of sebaceous hyperplasia [8].

In addition, Harwood et al. reported that exposure to azathioprine (AZA) induced a higher frequency of mutations in DNA MMR defect and SC development in vivo and in vitro, but the exact mechanism is unknown [7]. An experimental genetic Msh2 knockout mouse model provided evidence that AZA-treated Msh2(+/−) mice developed diffuse lymphomas lacking Msh2 expression and displaying MSI due to somatic inactivation of the functional Msh2 allele by loss of heterozygosity or mutation [9]. These results suggest that AZA played an important role in the carcinogenesis in our case because the patient took AZA for 2 years, starting 3 years after kidney transplantation. The evidence described above suggests that MTS might have been unmasked due to the influence of immunosuppressive agents.

The optimal method for preventing the development of neoplasms in transplant recipients with MTS remains unclear. Calcineurin inhibitors (CNI), such as cyclosporine A and tacrolimus, influence tumor progression via TGF-β1 and VEGF overexpression, and were shown to induce tumor invasion metastasis and angiogenesis in vivo and in vitro [10, 11]. These drugs also inhibit nucleotide excision repair (NER), another DNA repair mechanism, resulting in cutaneous carcinogenesis [12]. In comparison, mTOR inhibitors, including sirolimus and everolimus, decreased the incidence of cancer by blocking cell proliferation via mTOR suppression and the expression of inhibitors of cell cycle-controlling cyclin (e.g., p27kip1) [11]. These findings suggest a potential anti-carcinogenic effect if a patient is switched from a CNI to an mTOR inhibitor. In one reported case, changing from tacrolimus to sirolimus markedly decreased the formation of cutaneous neoplasms in a kidney transplant recipient with unrecognized MTS [13]. Further studies are needed to better understand the association between immunosuppressive agents and the development of neoplasms in MTS-positive transplant recipients.

Patients diagnosed with MTS and at-risk family members have increased risk of the development of multiple neoplasms. In fact, MSH2/MLH1 gene mutation carriers, as in our case, have a lifetime risk of colorectal cancer ranging from 22 to 74% [14]. Therefore, preventive cancer screening should be performed in these individuals as well as in patients with HNPCC, according to the guidelines for cancer surveillance. The guidelines proposed by the American College of Gastroenterology include the following recommendations: Colonoscopy for colorectal cancer at least every 2 years beginning between the ages of 20 to 25 years, endometrial biopsy and transvaginal ultrasound annually for endometrial cancer and ovarian cancer beginning between the ages of 30 to 35 years, esophagogastroduodenoscopy with gastric biopsy for gastric and duodenal cancer between the ages of 30 to 35 years, and treatment of *Helicobacter pylori* infection when found [14]. Accordingly, such cancer surveillance and genetic studies should be performed not only for the patient but also for at-risk family members, especially, in this case, her brother with colorectal cancer at the relatively young age of 45 years.

In conclusion, we report the case of kidney transplant recipient with SC concurrent with colon cancer due to MTS. The causes of the neoplasms in this case seem to be multifactorial. Whether the patient should be switched from a CNI to an mTOR inhibitor remains unclear; thus, further observation should be performed to prevent the development of neoplasms.

## Data Availability

The data that support the findings of this study are available from the corresponding author, YN, upon reasonable request.

## Abbreviations

AZA: Azathioprine
CNI: Calcineurin inhibitor
HNPCC: Hereditary nonpolyposis colorectal cancer
MMF: Mycophenolate mofetil
MMR: Mismatch repair
MSI: Microsatellite instability
mTOR: Mammalian target of rapamycin
MTS: Muir–Torre syndrome
NER: Nucleotide excision repair
SC: Sebaceous carcinoma
